# Suppressing migration and invasion of H1299 lung cancer cells by honokiol through disrupting expression of an HDAC6‐mediated matrix metalloproteinase 9

**DOI:** 10.1002/fsn3.1439

**Published:** 2020-02-06

**Authors:** Jih‐Tung Pai, Chia‐Yun Hsu, Yei‐San Hsieh, Tsung‐Yu Tsai, Kuo‐Tai Hua, Meng‐Shih Weng

**Affiliations:** ^1^ Division of Hematology and Oncology Tao‐Yuan General Hospital Ministry of Health and Welfare Taoyuan City Taiwan; ^2^ Department of Nutritional Science Fu Jen Catholic University New Taipei city Taiwan; ^3^ Department of Chest Surgery Tao‐Yuan General Hospital Ministry of Health and Welfare Taoyuan City Taiwan; ^4^ Department of Food Science Fu Jen Catholic University New Taipei City Taiwan; ^5^ Graduate Institute of Toxicology College of Medicine National Taiwan University Taipei Taiwan

**Keywords:** HDAC6, honokiol, Hsp90, hyperacetylation, matrix metalloproteinases (MMPs)

## Abstract

Metastasis is the crucial mechanism to cause high mortality in lung cancer. Degradation of extracellular matrix (ECM) by proteolytic enzymes, especially matrix metalloproteinases (MMPs), is a key process for promoting cancer cell migration and invasion. Therefore, targeting MMPs might be a strategy for lung cancer metastasis suppression. Honokiol, a biological active component of *Magnolia officinalis*, has been indicated to suppress lung cancer tumorigenesis through epigenetic regulation. However, the regulation of MMPs‐mediated migration and invasion by honokiol through epigenetic regulation in lung cancer is still a mystery. In the present study, the migration and invasion ability of H1299 lung cancer was suppressed by noncytotoxic concentrations of honokiol treatment. The proteolytic activity of MMP‐9, rather than MMP‐2, was inhibited in honokiol‐treated H1299 cells. Honokiol‐inhibited MMP‐9 expression was through promoting MMP‐9 protein degradation rather than suppressing transcription mechanism. Furthermore, the expression of specific histone deacetylases 6 (HDAC6) substrate, acetyl‐α‐tubulin, was accumulated after honokiol incubation. The disassociation of MMP‐9 with hyper‐acetylated heat shock protein 90 (Hsp90) was observed resulting in MMP‐9 degradation after honokiol treatment. Meanwhile, honokiol‐suppressed MMP‐9 expression and invasion ability of H1299 lung cancer cells was rescued by HDAC6 overexpression. Accordingly, the results suggested that the suppression of migration and invasion activities by honokiol was through inhibiting HDAC6‐mediated Hsp90/MMP‐9 interaction and followed by MMP‐9 degradation in lung cancer.

## Graphical abstract

The suppression of migration and invasion activities of honokiol was through inhibiting HDAC6‐mediated Hsp90/MMP‐9 interaction and followed by MMP‐9 degradation in lung cancer.

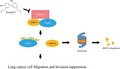

## 1 INTRODUCTION

Lung cancer is the leading cause of cancer‐related death worldwide (Torre et al., 2015). The high mortality rate of lung cancer is caused by metastasis which is a process of tumor cells from primary site to distant organs including cell motility, local cell invasion, intravasation, extravasation, and growth (Chambers, Groom, & MacDonald, 2002). The critical process of cancer cell metastasis is extracellular matrix (ECM) degradation. Numerous proteolytic enzymes involve in ECM degradation and promote cell invasion, such as matrix metalloproteinases (MMPs) (Gialeli, Theocharis, & Karamanos, 2011). Ectopic expressions of MMPs in different cancers are highly correlated with cancer cell invasion and poor prognosis (Deryugina & Quigley, 2006; Gong et al., 2016). MMP‐9 (gelatinase‐B) is the most important of MMPs which degrades the type IV collagen and is highly active in many cancer cells, such as breast, brain, prostate, and lung cancer (Alaseem et al., 2017; Gialeli et al., 2011; Gong et al., 2016; Nelson, Fingleton, Rothenberg, & Matrisian, 2000). Elevation of serum MMP‐9 expression is found in lung cancer patients than healthy people (Blanco‐Prieto et al., 2017). Furthermore, high activity of serum MMP‐9 and expression of MMP‐9 in the tumor tissue are significantly linked with lung cancer metastasis, tumor stage, and poor 5‐year overall survival rate (Gong et al., 2016). Suppression of lung cancer cell metastasis by down‐regulated activity and expression of MMP‐9 has been evaluated (Chao, Deng, Li, Liang, & Huang, 2017; Li et al., 2017). Therefore, targeting MMPs might suppress lung cancer metastasis and improve therapeutic outcome.

The MMP‐9 activity and expression are governed by transcription, post‐transcription, translation, and post‐translation mechanisms. Transcription factors, such as AP‐1, PEA3, and nuclear factor kappa B (NF‐κB), are well‐known to up‐regulate MMP‐9 gene expression (Yan & Boyd, 2007). Inhibition of these transcription factors‐mediated signaling pathways suppresses MMP‐9 expression and cancer cell metastasis. In addition to transcription regulation, disruption of MMP‐9 protein stability through post‐translation regulation is shown to suppress MMP‐9‐mediated metastasis. Numerous studies show that the chaperone protein, Hsp90, is involved in MMP‐2/9 activation by protein–protein interaction (Eustace et al., 2004; Stellas, El Hamidieh, & Patsavoudi, 2010). Suppression of cancer cells metastasis has been investigated through down‐regulation of Hsp90 expression and/or disruption of the interaction of Hsp90 with MMP‐2/9 (Kim et al., 2008; Stellas et al., 2010). Post‐translational modifications, such as phosphorylation and acetylation, are critical mechanism to control Hsp90 chaperone function (Scroggins et al., 2007; Sima & Richter, 2018). Induction of Hsp90 hyperacetylation to dissociate with its binding proteins is identified in HDACs inhibitor‐treated cancer cells (Kovacs et al., 2005; Liou, Hua, Hsu, & Weng, 2015; Park et al., 2008). Thus, repression of HDACs‐regulated Hsp90 function might be a strategy to repress cancer cell metastasis.

HDACs remove the acetyl group from histones or nonhistone protein at lysine residues and initiate the regulation of gene expression and protein stability (Cress & Seto, 2000). Eighteen human HDACs have been discovered and classified into four sub‐groups. Class I HDACs regulate histone acetylation status and govern the gene expression. Class II HDACs participate in deacetylation of nonhistone substrates and regulation of the protein stability. Aberrant expression of HDACs has been displayed the connection in tumor progression. The expressions of HDAC1 and HDAC5 exhibit a poor prognosis in lung and breast cancer, respectively (Li et al., 2016; Minamiya et al., 2011). Expression of HDAC6, a member of class II HDACs, promotes endothelial cell migration and angiogenesis by deacetylation of cortactin (Kaluza et al., 2011). Down‐regulation of E‐cadherin via HDACs is observed in smoking‐induced lung cancer migration and invasion (Nagathihalli, Massion, Gonzalez, Lu, & Datta, 2012). In addition, suppression of lung cancer invasion is perceived in HDAC inhibitor‐repressed MMP‐2/9 activity (Karthik, Sankar, Varunkumar, & Ravikumar, 2014; Liu, Chang, Chiang, & Hung, 2003). However, suppression of metastasis and expression of MMP‐2/9 are also detected in HDAC10 up‐regulation of cervical cancer cells (Song, Zhu, Wu, & Kang, 2013). Further evaluation of the relationship between HDAC and MMPs might benefit for cancer therapy and prevention.

Several evidences indicate that bioactive compounds from traditional Chinese medicines (TCMs) prevent cancer progression, such as breast, colon, and lung cancer (Arora et al., 2012; Hu, An, Wang, Chen, & Xu, 2016; You, An, Liang, & Wang, 2013). *Magnolia officinalis* plant, as known as houpu magnolia, has been used as TCM for many years in East and Southeast Asia countries. Honokiol, one of the bioactive components of *Magnolia officinalis*, has been validated possessing many activities, especially for antineoplastic properties (Arora et al., 2012). Recent evidences show that the antineoplastic properties of honokiol are through apoptosis and the induction of cell cycle arrest, the down‐regulation of epidermal growth factor receptor (EGFR) signaling pathway, the suppression of NF‐κB and signal transducers and activator of transcription 3 (STAT3) activation (Arora et al., 2012; Avtanski et al., 2014; Fan, Xue, Schachner, & Zhao, 2018; Tse, Wan, Shen, Yang, & Fong, 2005). Suppression of STAT3‐regulated epithelial‐to‐mesenchymal transition (EMT) leading to blockade breast cancer cell migration and invasion by honokiol has been uncovered (Avtanski et al., 2014). Inhibition of cancer cell metastasis by honokiol via EGFR‐ and vascular endothelial growth factor (VEGF)‐mediated signaling pathway has been noted in xenograft tumor model (Wen et al., 2009; Yang et al., 2017). Tumor necrosis factor‐α (TNF‐α)‐induced migration and MMP‐2 and MMP‐9 expression through ERK/NF‐κB signaling pathway are inhibited in honokiol‐treated rat aortic smooth muscle cells (Zhu, Wang, Hu, Li, & Hu, 2014). Furthermore, suppression of lung cancer cell growth by honokiol has been directed through class I HDACs inhibition (Singh, Prasad, & Katiyar, 2013). Suppression of HDAC6 activity via honokiol leads to disrupting EGFR and Hsp90 association following by EGFR degradation in lung cancer (Liou et al., 2015). Although the anticancer activity of honokiol has been confirmed, the relationship between inhibition of HDAC and suppression of MMP‐mediated migration and invasion by honokiol in lung cancer cells is still unclear. In the present study, we examined the antimigration and anti‐invasion effects of honokiol by HDAC‐mediated MMPs signaling pathway. Our results revealed that epigenetic mechanism‐regulated migration and invasion might be a target of honokiol. Disruption of MMP‐9 protein stability via honokiol was through HDAC6 activity inhibition, and following the suppression lung cancer cell migration and invasion.

## 2 MATERIALS AND METHODS

### 2.1 Reagents

Honokiol, MG132, and tubacin were obtained from Biomol/Enzo Life Sciences International, Inc. (Plymouth Meeting, PA, USA). Mouse anti‐MMP‐9, anti‐HDAC6, anti‐acetyl‐α‐tubulin, and anti‐acetyllysine antibodies were purchased from Cell Signaling Technology (Beverly, MA, USA). Mouse anti‐α‐tubulin, anti‐β‐actin, anti‐Hsp90, anti‐ubiquitin, and protein A/G plus agarose were gained from Santa Cruz Biotechnology (Santa Cruz, CA, USA).

### 2.2 Cell viability assays

H1299 lung cancer cell line was obtained from the American Type Culture Collection (Manassas, VA, USA). Cells were cultured in 5% serum‐containing RPMI‐1640 (Hyclone Laboratories, Logan, UT, USA) and incubated at 37℃ in 5% CO_2_ atmosphere. For cell viability analyses, H1299 cells (1 × 10^5^/well) were cultured in 96‐well plates and then stimulated with honokiol (0, 2.5, 5, 7.5 and 10 μM) for 24 hr. Thereafter, MTT assay was performed to evaluate cell viability.

For cell proliferation assay, trypan blue exclusion analysis was performed. Briefly, H1299 cells (1 × 10^6^/well) were plated in 6‐well dish and then stimulated with honokiol (0, 2.5, 5, 7.5, and 10 μM) for 24 hr. Afterward, cells were collected and trypan blue staining was used for cell counting.

### 2.3 Determination of cell cycle

Cell was cultured and synchronized. After 18 hr of synchronization, serum‐free RPMI medium was replaced by serum‐containing medium, and then, honokiol was treated. After 24 hr treatment, cells were stained with PI (propidium iodide, Sigma Chemical, St. Louis, MO, USA). The PI fluorescence was detected by FACScan laser flow cytometer (Beckman Coulter, Fullerton, CA, USA).

### 2.4 In vitro wound closure

In vitro wound closure experiment was modified from previous study (Hsieh, Liao, Chen, Pai, & Weng, 2017). Cells (1 × 10^6^ cells/well) were seeded in 6‐well cultured dish for 24 hr. Thereafter, wounded area was scratched and then incubated with medium containing with or without honokiol (0, 2.5, 5, 7.5, 10 μM) for 24 hr. Phase‐contrast microscopy was used to photograph the wound closure.

### 2.5 In vitro migration and invasion analyses

Cells were stimulated with honokiol (0, 2.5, 5, 7.5, and 10 μM) for 24 hr. Then, cells were seeded (1 × 10^5^ cells/well) on Boyden chamber (BD Bioscience, Bedford, MA, USA) in serum‐free medium for 24 hr. To assay the in vitro invasion assay, Matrigel‐coating pore polycarbonate filters were used and serum‐containing medium was added in the lower chamber. After 24 hr incubation, the invaded cells were fixed with methanol at 4℃ for 15 min. Cells were then stained with crystal violet, and the number of invade cells was counted. For evaluating in vitro migration activity, cells were carried out as described in vitro invasion assay without Matrigel coating.

### 2.6 Gelatin zymography assay

H1299 cells (6 × 10^5^ cells/well) were seeded in 10‐cm petri dish and subsequently incubated with serial concentrations of honokiol (0, 2.5, 5, 7.5, 10 μM) for 24 hr. Conditioned media were collected with nonreducing sample buffer and subjected to 8% SDS‐PAGE electrophoresis containing 0.1% gelatin. The gels were then washed twice with 2.5% Triton X‐100 and incubated with reaction buffer (40 mM Tris‐HCl with pH 8.0, 10 mM CaCl_2_, and 0.001% NaN_3_) at 37°C for 24 hr. After incubation, gel was stained with Coomassie Blue and destained with destaining buffer. The zones of gelatinolytic activity were presented as negative staining and were analyzed by density measurement software.

### 2.7 Western blot analyses

Cell lysates were harvested, quantitated, and electrophoresed via SDS‐PAGE. The transferred membranes were then blocked with 5% skim milk and hybridized with various antibodies. The protein expressions were analyzed by ECL reagent (GE Healthcare Bio‐Sciences, Pittsburgh, PA, USA) and then quantitated by a UVP BioSpectrum Imaging System.

### 2.8 Reverse transcription‐polymerase chain reaction (RT‐PCR)

The total RNA of honokiol‐treated H1299 cells was extracted by an RNA Mini Kit (Qiagen, Taipei, Taiwan). cDNAs were obtained by commercial cDNA reverse transcription kit (Invitrogen, Taipei, Taiwan). The MMP‐9 forward primer 5′‐CGAACTTTGACAGCGACAAG‐3′ and reverse primer 5′‐TCAAAGACCGAGTCCAGCTT‐3′ yielded an amplicon of 586 bp, while the sequence of GAPDH forward primer 5′‐TGAAGGTCGGAGTCAACGGGTGAGTT‐3′ and reverse primer 5′‐CATGTAGACCCCTTGAAGAGG‐3′ yielded an amplicon of 983 bp. The 35 cycles of amplification for MMP‐9 were 95℃ for 50 s, 59℃ for 45 s, and 72℃ for 60 s and 30 cycles of amplification for GAPDH were 94℃ for 50 s, 60℃ for 45 s, and 72℃ for 120 s. The 1.8% agarose gel was performed to separate PCR products, and SYBR Safe dye (Life Technologies, Taipei, Taiwan) was used for gel staining. Quantitation of MMP‐9 and GAPDH gene expression was administrated by UVP BioSpectrum Imaging System.

### 2.9 Immunoprecipitation

Immunoprecipitation experiments were modified from previous study (Yu et al., 2014). One milligram of cell lysates was co‐incubated with indicated antibody and protein A/G plus agarose at 4℃ for 18 hr. The immuno‐complexes were washed and then resuspended with protein loading buffer. Western blot analyses were performed to detect the indicated protein expressions.

### 2.10 HDAC6‐overexpressive plasmid transfection

H1299 cells were seeded and grew to about 70% confluence, and then, the empty vector or HDAC6 plasmid (constructed by Prof. Hua) was transfected by GenMuteTM cDNA Transfection Reagent (SignaGen Laboratories, Ijamsville, MD, USA). After 24 hr transfection, the expressions of indicated protein were detected by Western blotting.

### 2.11 Statistical analyses

All results were performed at least three independent experiments and represented by the mean ± *SD*. The significant differences were calculated by one‐way ANOVA and followed by post hoc tests. The statistically significant was considered when *p*‐value was <.05.

## 3 RESULTS

### 3.1 Honokiol inhibited lung cancer cell migration and invasion

**Figure 1:**
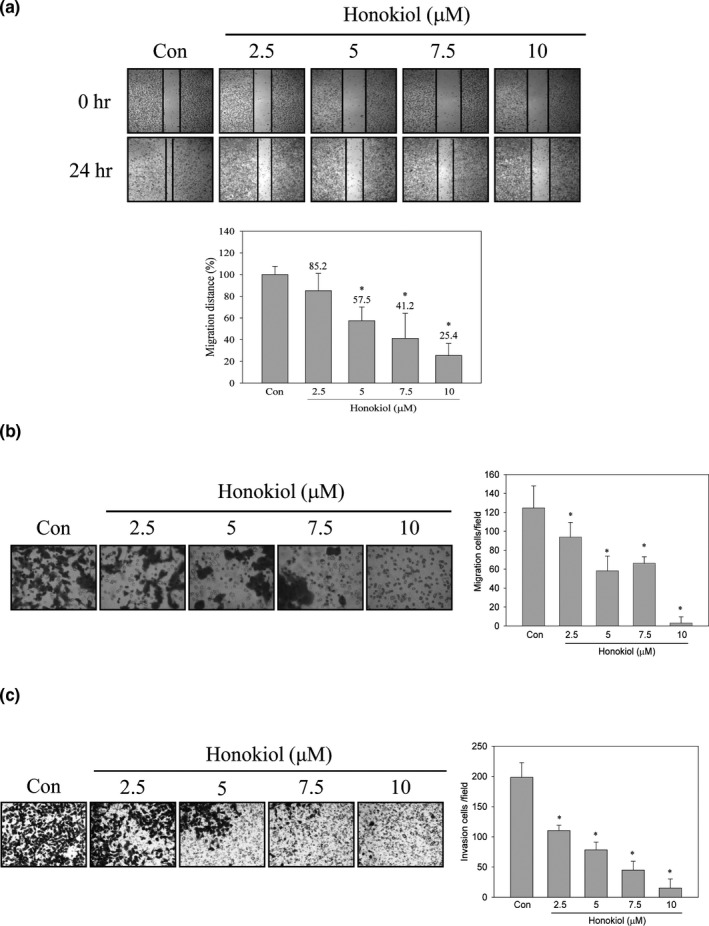
Effects of honokiol on migration and invasion of H1299 cells. H1299 cells were treated with honokiol (0, 2.5, 5, 7.5, 10 μM) for 24 hr, and (a) wound healing, (b) transwell migration, and (c) invasion analyses were performed. Data were represented by the mean ± SD of triplicate samples. Significant difference was compared with the control group (*, *p* < .05)

To understand the antimigration and anti‐invasion activity of honokiol, highly invasive lung cancer H1299 cells were treated with serial concentrations of honokiol (0, 2.5, 5, 7.5, 10 μM) for 24 hr. After treatment, in vitro wound healing, migration, and invasion analyses were performed. The wound healing ability of H1299 cells was dose‐dependently suppressed by honokiol (Figure 1a). The percentage of wound closure ability was decreased by approximately 15%, 43%, 59%, and 75% in serial concentrations of honokiol treatment. Furthermore, in vitro migration assays revealed that the numbers of migration cells were decreased after honokiol treatments (Figure 1b). In addition, the invasion ability of H1299 cells was also repressed by honokiol in a dose‐dependent mode (Figure 1c).

**Figure 2:**
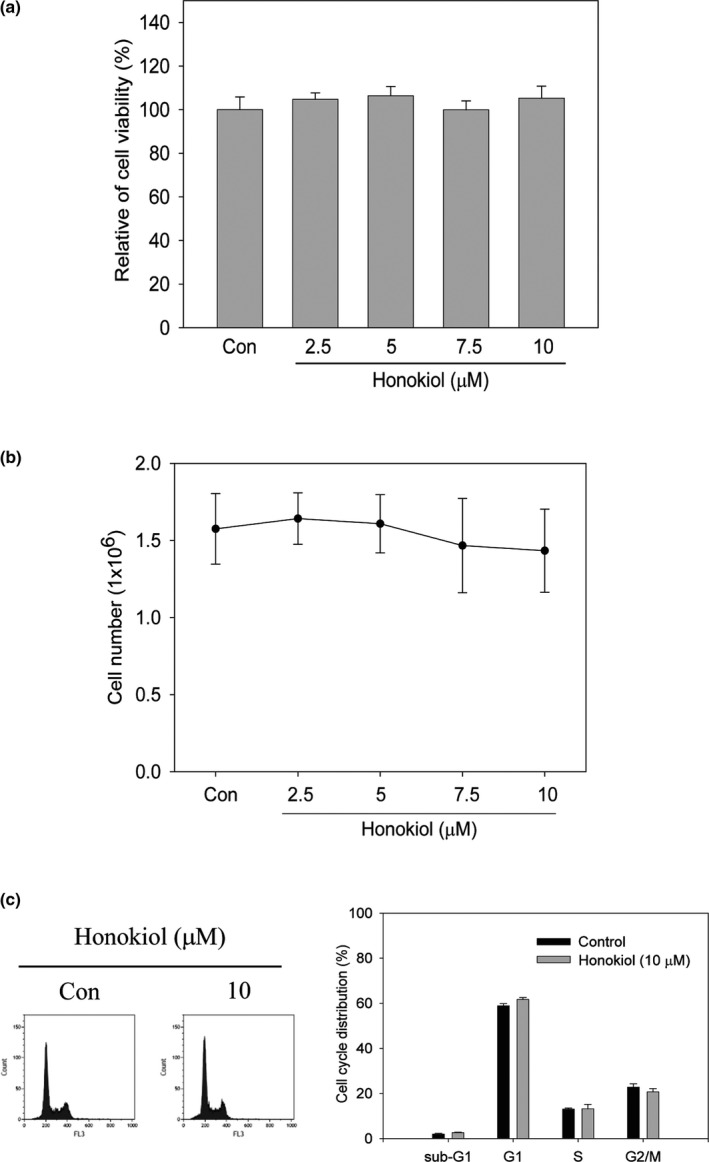
Effects of honokiol on cell viability and cell cycle distribution in H1299 cells. H1299 cells were cultured and treated with honokiol (0, 2.5, 5, 7.5, 10 μM) for 24 hr. (a) cell viability and (b) cell proliferation were detected by MTT assay and trypan blue exclusion assay, respectively. (c) H1299 cells were incubated with 10 μM of honokiol for 24 hr. Thereafter, cells were harvested and flow cytometry was performed to determine cell cycle distribution. Data were represented by the mean ± SD of triplicate samples. Significant difference was compared with the control group (*, *p* < .05)

To exclude the possibility that suppressing migration and invasion activities of honokiol resulted from proliferation inhibition, cell viability and proliferation analyses were examined. As shown in Figure 2, cell viability was no significant difference between control and honokiol‐treated cells (Figure 2a). Meanwhile, the significant changes in the cell number between control and honokiol‐treated cell by trypan blue exclusion assay were not observed (Figure 2b). Further examining the effect of honokiol on cell cycle distribution, the results showed that cell cycle distribution with 10 μM of honokiol treatment was not affected (Figure 2c). According to these results, cell migration and invasion abilities of H1299 were suppressed by honokiol under noncytotoxic concentration.

### 3.2 Honokiol inhibited the proteolytic activity and expression of MMP‐9

**Figure 3:**
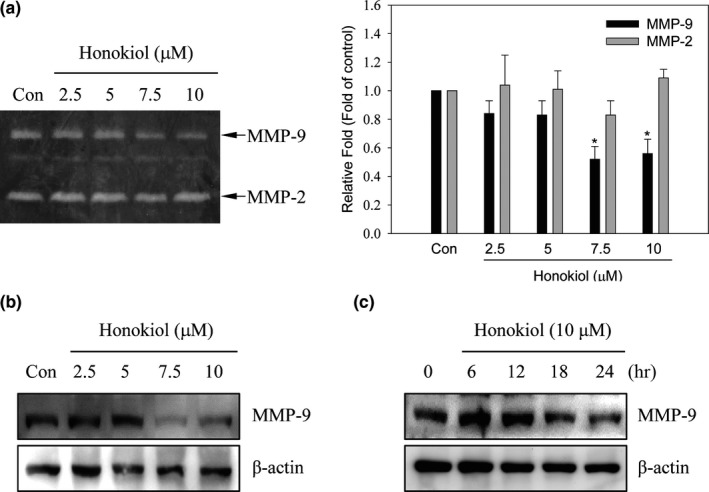
Effects of honokiol on MMP‐9 activity and protein expression. H1299 cells were cultured and treated with honokiol (0, 2.5, 5, 7.5, 10 μM) for 24 hr. After treatment (a), the culture media were collected and the activity of MMP‐2 and MMP‐9 was implemented by gelatin zymography analyses. After (b) a serial dosage of honokiol (0, 2.5, 5, 7.5, 10 μM) treatment for 24 hr or (c) the addition of 10 μM honokiol for different time interval, cell lysates were harvested and the expression of MMP‐9 protein was detected by Western blotting method

MMP‐2 and MMP‐9, two important ECM‐degrading enzymes, participate in cancer cell migration and invasion (Gialeli et al., 2011). We next evaluated the inhibition effects of honokiol in proteolytic activity and expression of MMP‐2 and MMP‐9. The results disclosed that the proteolytic activity of MMP‐9, rather than MMP‐2, was inhibited via honokiol (Figure 3a). The proteolytic activity of MMP‐9 was decreased about 50% after 7.5 or 10 μM of honokiol treatment. Besides, the expression of MMP‐9 protein was inhibited after 7.5 and 10 μM of honokiol treatment for 24 hr (Figure 3b). Meanwhile, down‐regulation of MMP‐9 expression was perceived after 18 hr of honokiol treatment (Figure 3c).

### 3.3 Honokiol‐suppressed MMP‐9 expression was through the regulation of ubiquitin–proteasome system

**Figure 4:**
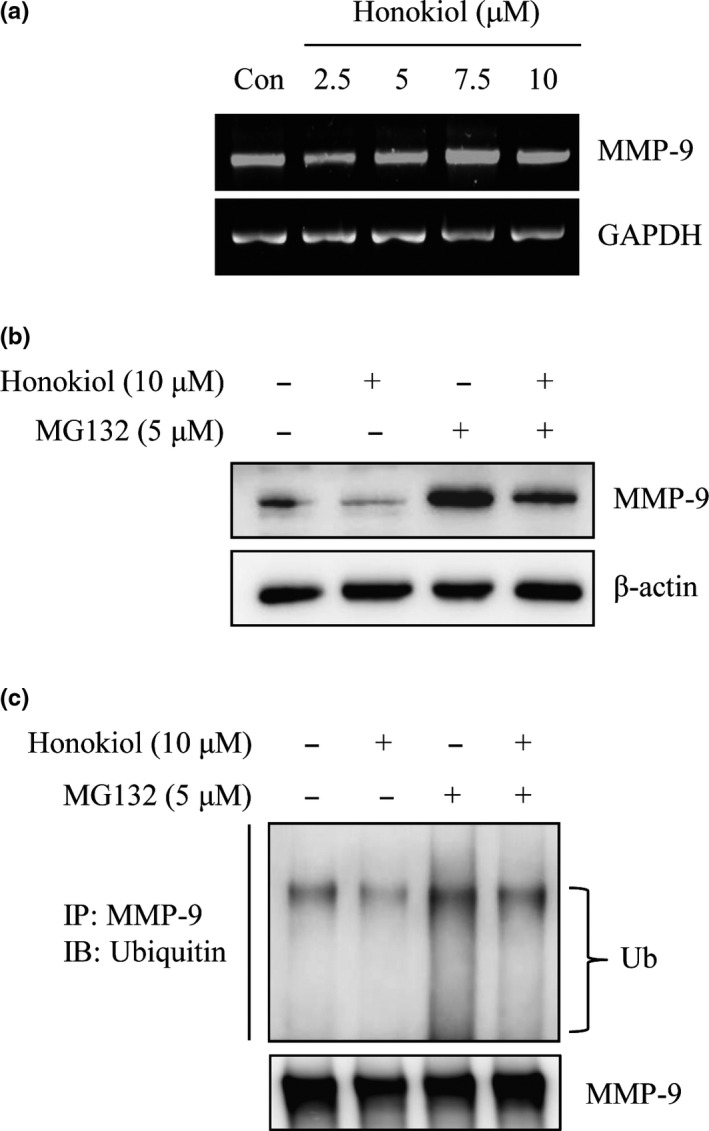
Down‐regulated MMP‐9 expression by honokiol through the degradation of ubiquitin/proteasome system in H1299 cells. (a) H1299 cells were treated with honokiol (0, 2.5, 5, 7.5, 10 μM) for 24 hr, the total mRNAs were harvested, and the mRNA level of MMP‐9 was detected by RT‐PCR assay. (b) H1299 cells were treated with or without 5 μM MG132 for 30 min before 10 μM honokiol incubation for 24 hr. After treatment, the expression of MMP‐9 protein was assayed by Western blotting or (c) the ubiquitination of MMP‐9 was analyzed by immunoprecipitation analysis with an anti‐MMP‐9 antibody. The immunoprecipitates were then analyzed the expression of ubiquitin by Western blotting

To evaluate the mechanism of honokiol‐inhibited MMP‐9 expression, the expression of MMP‐9 mRNA in honokiol‐treated cells was inspected. As shown in Figure 4a, the expression of MMP‐9 mRNA was not affected after honokiol treatments (Figure 4a). Since down‐regulated MMP‐9 via honokiol was not through transcriptional regulation, the role of honokiol on the inhibition of MMP‐9 expression via protein degradation regulation was investigated. Cells were treated with or without 5 μM of MG132, a proteasome inhibitor, for 30 min before honokiol treatment, and the expression of MMP‐9 was then detected by Western blot. The results revealed that honokiol‐inhibited MMP‐9 expression was reversed by MG132 pretreatment (Figure 4b). To further examine the poly‐ubiquitylation of MMP‐9 in honokiol‐treated cells, immunoprecipitation of MMP‐9 was performed and poly‐ubiquitin was determined by Western blots. Accumulation of poly‐ubiquitin was clearly detected in co‐treatment with MG132 and honokiol (Figure 4c). These results exhibited that honokiol‐inhibited MMP‐9 expression was through ubiquitin–proteasome degradation rather than transcriptional inhibition.

### 3.4 Honokiol‐suppressed MMP‐9 expression was through the inhibition of HDAC6/Hsp90 signaling pathway

**Figure 5:**
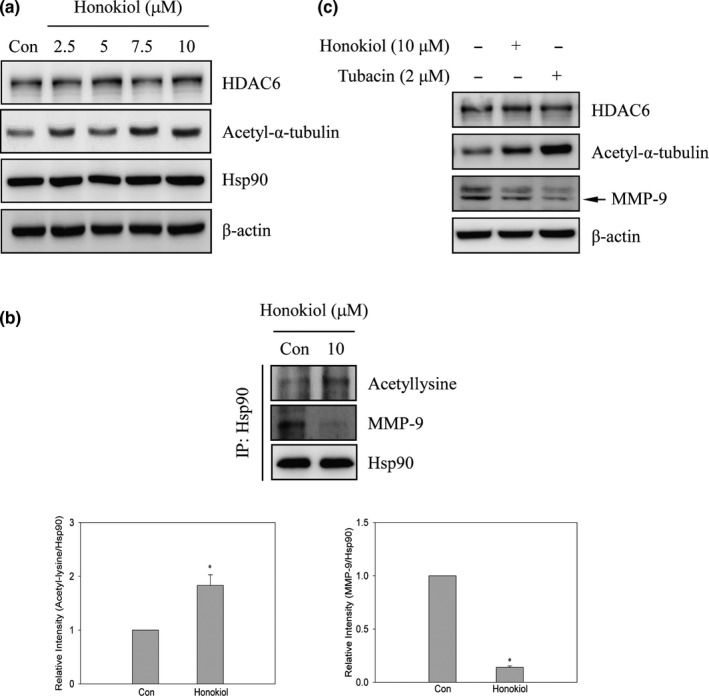
The disruption of MMP‐9/Hsp90 interaction by honokiol through the inhibition of HDAC6 activity in H1299 cells. (a) H1299 cells were treated with honokiol (0, 2.5, 5, 7.5, 10 μM) for 24 hr. After treatment, Western blot analyses were used to identify the expression of HDAC6, acetyl‐α‐tubulin, Hsp90, and β‐actin. (b) H1299 cells were treated with 10 μM of honokiol for 24 hr, and then, immunoprecipitation analysis was performed. The immunoprecipitates of anti‐acetyllysine and MMP‐9 were then revealed by Western blotting. (c) H1299 cells were incubated with 2 μM of tubacin or 10 μM of honokiol for 24 hr. Afterward, cells were collected and the expression of HDAC6, acetyl‐α‐tubulin, and MMP‐9 were determined by Western blot analyses. Data were represented by the mean ± SD of triplicate samples. Significant difference was compared with the control group (*, *p* < .05)

Disruption of Hsp90 function decreases target protein stability and prompting target protein degradation (Liou et al., 2015; Trepel, Mollapour, Giaccone, & Neckers, 2010). Inducing Hsp90 acetylation through honokiol‐inhibited HDAC6 following disrupting Hsp90’s chaperone function resulting in prompting target protein degradation has been demonstrated in A549 cells (Liou et al., 2015). To understand whether honokiol‐suppressed migration and invasion was through the inhibition of HDAC6/Hsp90 signaling pathway in our system, the expression of HDAC6‐specific substrate, acetyl‐α‐tubulin, was inspected. The results revealed that the expression of acetyl‐α‐tubulin, rather than Hsp90 and HDAC6, was increased after honokiol treatment (Figure 5a). Immunoprecipitation analysis also indicated that the relative expression of Hsp90’s acetyllysine was increased approximately 2‐folds after honokiol treatment (Figure 5b). In addition, the level of bound MMP‐9 with Hsp90 was dramatically diminished after honokiol incubation (Figure 5b). These results implied that honokiol‐induced MMP‐9 degradation might be through HDAC6/Hsp90 signaling pathway suppression. To further demonstrate the role of HDAC6/Hsp90‐mediated MMP‐9 regulation, tubacin, a specific pharmacological HDAC6 inhibitor, was implemented to evaluate the relationship between HDAC6 and MMP‐9. The outcomes displayed that the expression of acetyl‐α‐tubulin was dramatically increased in tubacin‐treated cells. Meanwhile, down‐regulation of MMP‐9 was also found after tubacin treatment (Figure 5c). Honokiol‐inhibited MMP‐9 expression exhibited similar results as displayed in tubacin‐treated cells (Figure 5c).

### 3.5 Honokiol‐suppressed MMP‐9‐regulated invasion was rescued by the overexpression of HDAC6

**Figure 6:**
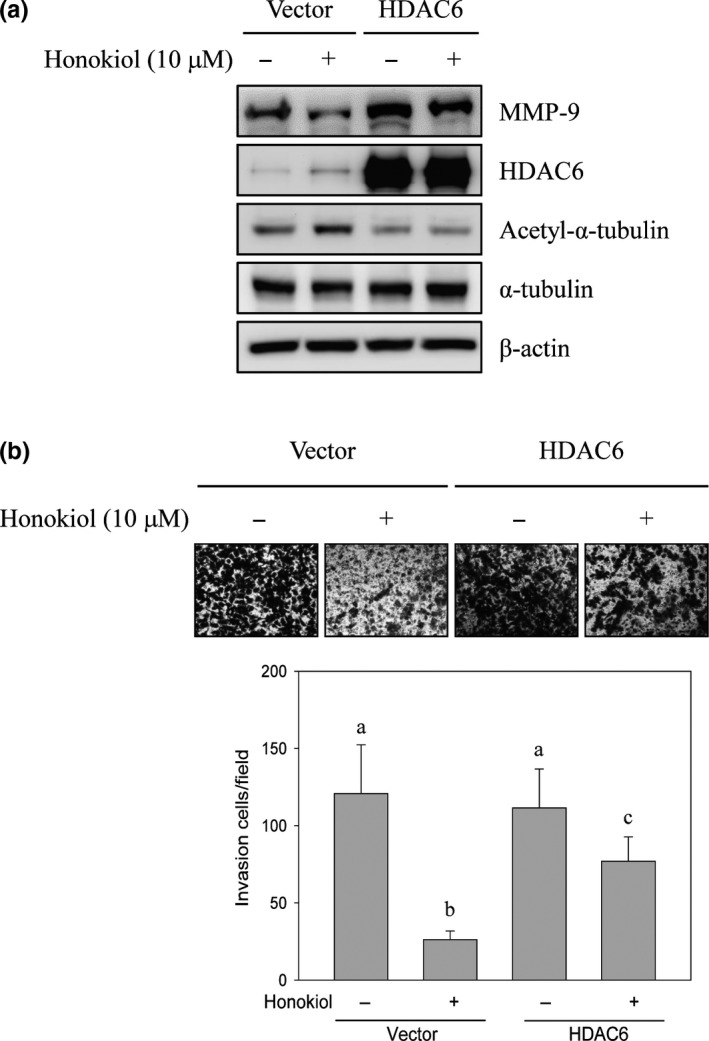
Ectopic expression of HDAC6 reversed honokiol‐suppressed H1299 cell invasion. H1299 cells were transiently transfected with empty vector or HDAC6 plasmid. After transfection, cells were treated with or without 10 μM of honokiol for 24 hr. (a) Cells were subsequently collected, and Western blot analyses were evaluated with indicated antibodies. (b) In vitro invasion activity of H1299 cells was estimated. Data were represented by the mean ± SD of triplicate samples. Different lowercase letters (a−c) indicated statistical differences among group (*p* < .05), and no difference (*p* > .05) was shown in the same letter

To further explore the role of HDAC6 in honokiol‐suppressed MMP‐9 expression, overexpression of HDAC6 system was conducted. Cells were transfected with empty vector or HDAC6‐overexpressive plasmid before honokiol treatment, and then, Western blot analyses were performed. After HDAC6‐overexpressive plasmid transfection, the expression of HDAC6 was dramatically increased and the protein level of acetyl‐α‐tubulin was decreased comparing with empty vector transfection. Honokiol‐induced acetyl‐α‐tubulin expression was suppressed in HDAC6‐transfected cells. Meanwhile, down‐regulation of MMP‐9 expression by honokiol was rescued by the overexpression of HDAC6 (Figure 6a). Thereafter, HDAC6‐mediated honokiol‐repressed invasion was inspected. The numbers of invasion cells were suppressed by honokiol in empty vector transfection. However, honokiol‐suppressed cell invasion was reversed by overexpression of HDAC6 (Figure 6b).

## 4 DISCUSSION

Degradation of the extracellular matrix is critical step for the malignant tumor migration and invasion. Matrix metalloproteinases, particularly MMP‐2 and MMP‐9, are crucial molecules in tumor development, progression, and metastasis (Alaseem et al., 2017; Deryugina & Quigley, 2006; Gong et al., 2016; Lagarrigue et al., 2010; Miao et al., 2017). Therefore, targeting MMP‐2 and/or MMP‐9 might provide a strategy for cancer treatment and prevention. In the present study, honokiol, an active compound from *Magnolia officinalis* plant, suppressed lung cancer cell migration and invasion via the inhibition of MMP‐9 activity, rather than MMP‐2. Disruption of MMP‐9 protein associated with Hsp90 and following by MMP‐9 degradation leading to the repression of migration and invasion activity via honokiol might result from HDAC6 inhibition in lung cancer cells.

Cancer metastasis progression is key step to the leading cause of cancer‐related death in lung cancer, especially when most patients are diagnosed with a later stage. Therefore, suppression of metastasis or prevention of micrometastasis is important for improving the survival rate in lung cancer patients. Metastasis is a complicated process which involves cell migration, local invasion, intravasation and blood circulation, extravasation, and growth at distant organs (Valastyan & Weinberg, 2011). During metastasis progress, proteolytic enzymes which degrade ECM for tumor cell dissemination are essential and critical for developments. Although plenty of proteinase genes have been evaluated, MMPs, especially MMP‐2 and MMP‐9, are importantly associated with metastatic processes (Alaseem et al., 2017). Not only do MMPs in lung tumorigenesis provide cancer cell dissemination but also contribute to formation of the complex microenvironment promoting malignant transformation in lung tissue. Aberrational expression of MMPs has been associated with lung cancer (Blanco‐Prieto et al., 2017; Gong et al., 2016). Evaluation of MMPs concentration of serum samples between lung cancer patients and healthy population indicated that the high expression of MMP‐1, MMP‐7 and MMP‐9 is noticed in lung cancer patients and MMP‐9 expression can discriminate early stage of lung cancer from healthy individuals (Blanco‐Prieto et al., 2017). Furthermore, the analysis of the correlation of tumor stage and MMP‐9 expressions reveals that high expression of MMP‐9 is found more in stage III and IV of lung cancer than stage I and II (El‐Badrawy, Yousef, Shaalan, & Elsamanoudy, 2014). High expression of MMP‐9 is also identified in lung cancer patients with low 5‐year survival rate (Zheng et al., 2010). Therefore, targeting MMPs, especially MMP‐9, might blockade lung cancer metastasis and improve survival rate. Inhibition of MMP‐9‐mediated lung cancer migration and invasion via honokiol was evaluated in the present study, and the migration and invasion activity of H1299 lung cancer cells was suppressed under the noncytotoxic concentration of honokiol treatments (Figures 1 and 2). Furthermore, the activity of MMP‐9, rather than MMP‐2, was suppressed by honokiol treatments (Figure 3a). Inhibition of MMP‐9 expression was also detected after 7.5 and 10 μM of honokiol treatments (Figure 3b). Meanwhile, down‐regulation of MMP‐9 expression was found in honokiol treatments with 18 hr incubation (Figure 3c). These results implied that antimigration and anti‐invasion activity of honokiol might be through MMP‐9 down‐regulation.

To address the mechanism of honokiol‐suppressed MMP‐9 expression, MMP‐9 mRNA expression in honokiol‐treated cells was evaluated. Figure 4a showed that the MMP‐9 mRNA expression was unaffected by honokiol treatment (Figure 4a). To further evaluate whether down‐regulation of MMP‐9 protein expression by honokiol was through promoting protein degradation, proteasome inhibitor MG132 was administrated to confirm the issue. As shown in Figure 4b, pretreatment with MG132 before honokiol incubation was reversed honokiol‐suppressed MMP‐9 protein expression. In addition, the ubiquitination of expressed MMP‐9 was also examined by immunoprecipitation assay. The outcomes revealed that the poly‐ubiquitin of MMP‐9 was dramatically increased in MG132 and honokiol co‐treatment cells (Figure 4c). The results indicated that down‐regulation of MMP‐9 protein expression by honokiol might be thru disruption of MMP‐9 protein stability, rather than transcriptional suppression. Recent study indicates that disruption of the interaction of Hsp90 and MMP‐2 and MMP‐9 results in metastasis suppression in breast cancer (Stellas et al., 2010). Moreover, the protection of MMP‐2 from the degradation in tumor cells by interacting with Hsp90α has been demonstrated (Song et al., 2010). Meanwhile, the suppression of NF‐κB‐dependent MMP‐9 expression has been discovered in Hsp90 inhibitor‐prevented cerebral ischemic stroke (Qi et al., 2015). The present results suggested that honokiol‐inhibited MMP‐9 expression might be through post‐translational regulation. Therefore, promoting MMPs protein degradation by the disruption of Hsp90 chaperone might be the target of honokiol in the present model.

The function of chaperone protein Hsp90 involves in the maturation and stabilization of target protein. Ectopic expression of Hsp90 in tumor cells protects serial of mutated and overexpressed oncoproteins from degradation (Kovacs et al., 2005; Park et al., 2008; Trepel et al., 2010). Disruption of Hsp90 function has been indicated to destabilize and degrade of VEGFR, EGFR, glucocorticoid receptor, and MMPs protein resulting in tumorigenesis suppression (Agyeman et al., 2016; Kim et al., 2008; Kovacs et al., 2005; Liou et al., 2015; Park et al., 2008; Sims, McCready, & Jay, 2011). Post‐translational modifications, such as phosphorylation and acetylation, are the crucial mechanisms to regulate Hsp90 chaperone function. Acetylation modification of Hsp90 is regulated by histone acetyltransferase (HATs)/HDACs systems and has been demonstrated to participate in tumorigenesis and metastasis (Kovacs et al., 2005; Park et al., 2008; Scroggins et al., 2007; Sima & Richter, 2018). Induction of Hsp90 acetylation has been detected in HDACs suppression models, especially in HDAC6 suppression (Kovacs et al., 2005; Park et al., 2008). A recent study shows that hyperacetylation of Hsp90 disrupts EGFR maturation leading to EGFR degradation via honokiol is through the direct suppression of HDAC6 activity, rather than down‐regulation of HDAC or Hsp90 protein (Liou et al., 2015). Accordingly, the down‐regulation of MMP‐9 expression by honokiol thru the disruption of HDAC6‐mediated Hsp90 chaperone function was hypothesized. To address this hypothesis, the role of HDAC6 in honokiol‐suppressed MMP‐9 expression in lung cancer cells was assessed. As shown in Figure 5a, the expression of HDAC6 and Hsp90 was not affected by honokiol treatments. However, the expression of acetyl‐α‐tubulin, a well‐known HDAC6 direct substrate, was increased after honokiol treatments. The results were consistent with the previous study since the observation of honokiol as a direct HDAC6 inhibitor, rather than the down‐regulated HDAC6 expression (Liou et al., 2015). However, our current study could not rule out the role of sirtuin 2 (SIRT2), another α‐tubulin acetylation enzyme, in honokiol‐induced acetyl‐α‐tubulin expression (Eshun‐Wilson et al., 2019). Further evaluation of the role of SIRT2 in honokiol‐induced α‐tubulin acetylation is essential.

To further verify whether down‐regulation of MMP‐9 expression via honokiol was through the regulation of HDAC6‐mediated Hsp90 acetylation, immunoprecipitation analysis was performed. The outcomes exposed that the hyperacetylation modification of Hsp90 was increased in honokiol‐treated cells. Interestingly, the level of bound MMP‐9 with Hsp90 was dramatically diminished after honokiol treatment (Figure 5b). Specific HDAC6 inhibitor, tubacin, was administrated to understand the role of HDAC in MMP‐9 expression. The consequences showed that the expression of acetyl‐α‐tubulin was dramatically increased in tubacin‐treated cells. Meanwhile, the expression of MMP‐9 was also suppressed after tubacin incubation (Figure 5c). Down‐regulation of MMP‐9 expression by honokiol was similar with tubacin‐treated cells (Figure 5c). To further understand whether suppression of HDAC6‐regulated MMP‐9 expression participates in the migration and invasion of honokiol‐repressed lung cancer cells, overexpression of HDAC6 was performed. As shown in Figure 6a, ectopic expression of HDAC6 and low expression of acetyl‐α‐tubulin was detected in HDAC6 plasmid‐transfected cells. Meanwhile, the expression of honokiol‐induced acetyl‐α‐tubulin was repressed by HDAC6 overexpression. In addition, honokiol‐inhibited MMP‐9 expression was rescued in HDAC6‐overexpressed lung cancer cells (Figure 6a). Furthermore, the invasion of honokiol‐repressed lung cancer cells was reverted by overexpressed‐HDAC6 (Figure 6b). Accordingly, the results designated that the stimulation of MMP‐9 protein instability and degradation via honokiol was triggered by inhibiting the function of HDAC6‐mediated Hsp90 chaperone and followed by the suppression of lung cancer cell migration and invasion.

Hyperacetylation sites of Hsp90 have been inspected, and at least 11 acetylation residues of Hsp90 are designated (Yang et al., 2008). The disassociation of MMP‐2 with Hsp90 is induced by K69 acetylation on Hsp90 and results in the suppression of breast cancer invasion (Yang et al., 2008). Hyperacetylation K294 residue of Hsp90 attenuates the affinity of the target protein, such as ErbB2, p53, and androgen receptor, with Hsp90 and following cell viability inhibition (Scroggins et al., 2007). Thus, hyperacetylation at the different sites on Hsp90 has different binding client proteins and biological functions. The present study revealed that honokiol inhibited MMP‐9 activity, rather than MMP‐2 (Figure 3a). Therefore, we speculated that honokiol‐induced hyperacetylation of Hsp90 might be at the site of K294, rather than K69 site. However, more experiments required to be conducted to address the subject. Furthermore, it is well‐known that Hsp90 is a specific substrate of HDAC6 (Kovacs et al., 2005; Sima & Richter, 2018). The induction of Hsp90 hyperacetylation by suppression HDAC6 activity has been demonstrated to regulate Hsp90’s function and destabilize several Hsp90 target proteins (Kovacs et al., 2005; Park et al., 2008; Scroggins et al., 2007; Yang et al., 2008). However, many studies also unveiled that Hsp90 hyperacetylation is induced by other non‐HDAC6 inhibitor treatments (Furumai et al., 2002; Joshi et al., 2015). Recent studies also demonstrated that not only HDAC6 activity but also class I HDACs activity are suppressed by honokiol (Liou et al., 2015; Singh et al., 2013). Therefore, honokiol‐induced Hsp90 hyperacetylation through class I HDACs suppression could not be ruled out in this study.

**Figure 7:**
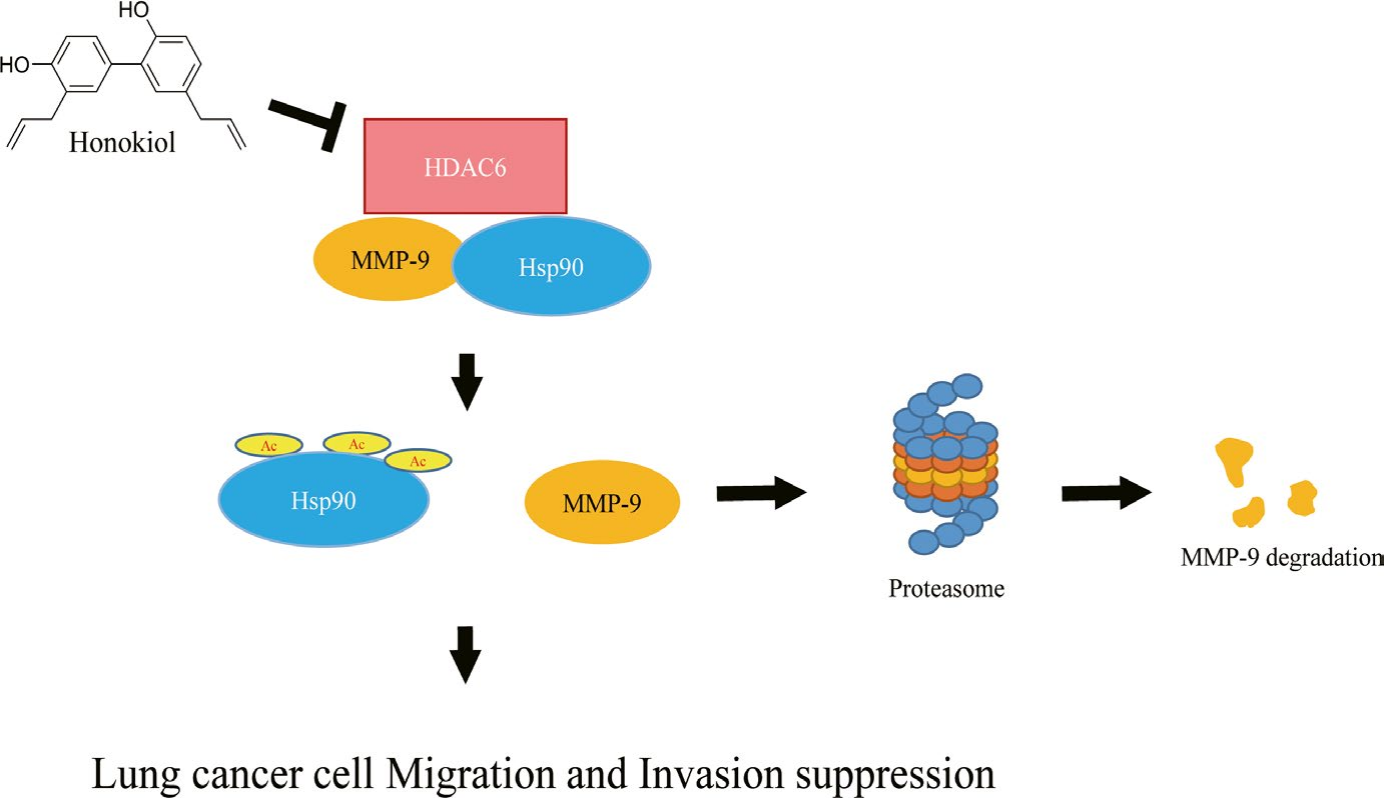
Schematic illustration of the antimigration and anti‐invasion mechanisms of honokiol on H1299 lung cancer cells

The promotion of cancer metastasis by MMP‐9 through EGFR‐mediated signaling pathway has been addressed (Cowden Dahl et al., 2008; Yang et al., 2017). The suppression of cancer cell metastasis thru the down‐regulation of EGFR‐activated MMP‐9 has been presented (Chung et al., 2017; Lin et al., 2017; Tse et al., 2005; Yang et al., 2017). Honokiol has been verified to inhibit the EGFR‐mediated signaling pathway through activity suppression and protein degradation (Liou et al., 2015; Yang et al., 2017). Accordingly, honokiol‐down‐regulated MMP‐9 expression might be possible through the repression of EGFR‐mediated signaling pathway. Nevertheless, EGFR‐activated MMP‐9 expression is through transcriptional factor‐drove MMP‐9 gene expression, such as NF‐κB or Sp‐1, but not the regulation of post‐translational mechanism (Lin et al., 2017; Yang et al., 2017). Hence, down‐regulated MMP‐9 expression thru the suppression of EGFR‐mediated signaling by honokiol was excluded in our system. The results demonstrated that the metastasis suppression in lung cancer cell by honokiol might be through epigenetic regulation of MMP‐9 expression via ubiquitin/proteasome degradation (Figure 7). The outcomes suggested that honokiol might be a potential chemoprevention agent for lung cancer metastasis through epigenetic regulation.

## CONFLICT OF INTEREST

The authors declare that they do not have any conflict of interest.

## ETHICAL APPROVAL

This study does not involve any human or animal testing.
